# Single-Chip Fully Integrated Direct-Modulation CMOS RF Transmitters for Short-Range Wireless Applications

**DOI:** 10.3390/s130809878

**Published:** 2013-08-02

**Authors:** Munir M. El-Desouki, Syed Manzoor Qasim, Mohammed BenSaleh, M. Jamal Deen

**Affiliations:** 1 King Abdulaziz City for Science and Technology (KACST), Riyadh 11442, Saudi Arabia; E-Mails: mqasim@kacst.edu.sa (S.M.Q.); mbensaleh@kacst.edu.sa (M.B.); 2 Department of Electrical and Computer Engineering, McMaster University, Hamilton, ON, Canada; E-Mail: jamal@mcmaster.ca

**Keywords:** CMOS, direct-modulation, phase-locked loop (PLL), radio frequency (RF), short-range, voltage-controlled oscillator (VCO), wireless transmitter

## Abstract

Ultra-low power radio frequency (RF) transceivers used in short-range application such as wireless sensor networks (WSNs) require efficient, reliable and fully integrated transmitter architectures with minimal building blocks. This paper presents the design, implementation and performance evaluation of single-chip, fully integrated 2.4 GHz and 433 MHz RF transmitters using direct-modulation power voltage-controlled oscillators (PVCOs) in addition to a 2.0 GHz phase-locked loop (PLL) based transmitter. All three RF transmitters have been fabricated in a standard mixed-signal CMOS 0.18 μm technology. Measurement results of the 2.4 GHz transmitter show an improvement in drain efficiency from 27% to 36%. The 2.4 GHz and 433 MHz transmitters deliver an output power of 8 dBm with a phase noise of −122 dBc/Hz at 1 MHz offset, while drawing 15.4 mA of current and an output power of 6.5 dBm with a phase noise of −120 dBc/Hz at 1 MHz offset, while drawing 20.8 mA of current from 1.5 V power supplies, respectively. The PLL transmitter delivers an output power of 9 mW with a locking range of 128 MHz and consumes 26 mA from 1.8 V power supply. The experimental results demonstrate that the RF transmitters can be efficiently used in low power WSN applications.

## 1. Introduction

**Figure 1.**
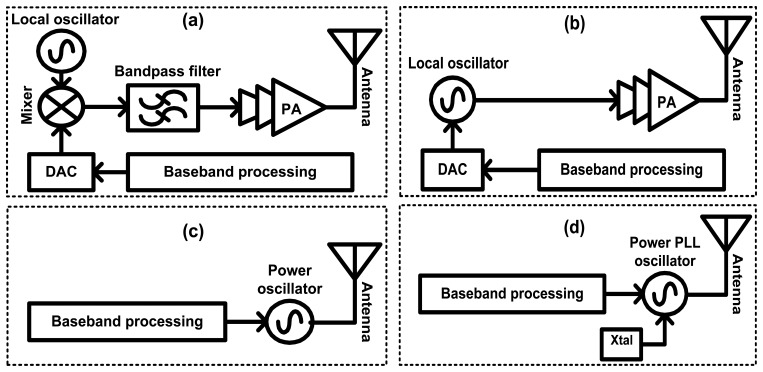
(**a**) Basic block diagram of a direct-modulation transmitter. (**b**) Simplified transmitter. (**c**) Single block transmitter using PVCO. (**d**) Single block transmitter using PVCO and PLL.

Short-range wireless transceivers are widely used in emerging low-power applications such as wireless sensor networks (WSNs), wireless body area networks (WBANs), and biomedical implantable electronic systems [1,2]. These applications have very strict requirements on the size, weight, cost and power consumption of the system and they are a special class of radio-frequency integrated circuits (RFICs) [3–5]. Therefore, having a simple design with minimal building blocks becomes an attractive approach for these applications [6]. The conventional architecture of a narrow-band direct-modulation transmitter is shown in Figure 1a. It includes a digital-to-analog converter (DAC), a mixer, a local oscillator, a band-pass filter and a power amplifier (PA). After the digital information is converted to an analog signal through the DAC, it is upconverted by the mixer to the carrier that is generated by the local oscillator. The signal is then filtered to remove harmonics generated by the mixer, after which its power is boosted by the PA. Figure 1b shows a first step to reducing the building blocks of the transmitter by using a voltage-controlled oscillator (VCO) to perform direct-frequency modulation (FM). By further simplifying and removing the DAC, direct-frequency shift keying (FSK) or Gaussian frequency shift keying (GFSK) can also be applied [7]. Figure 1c further reduces the blocks to only one, which is a power voltage-controlled oscillator (PVCO). Similar power oscillators are described in [8–11]; however none of these were fabricated using a standard CMOS process.

This paper discusses the feasibility of using CMOS PVCO based fully integrated, direct modulation transmitters in RF transceivers with comparable performance to other technologies. The main drawback of the architecture shown in Figure 1c is the frequency drift since the PVCO is used in open loop. This problem can be solved by the architecture in [12], however, it was not a single-block design and required a PA to boost the output signal. Therefore, a new topology based on phase-locked loop (PLL) is used as shown in Figure 1d. This improves the architecture shown in Figure 1c by implementing a PLL along with the PVCO to stabilize the frequency against any drift that might occur due to temperature or supply voltage variations, for instance. This paper also presents the design implementation and measurement results of 2.4 GHz and 433 MHz PVCO based transmitters and 2.0 GHz PLL based transmitter, respectively. Due to the limited availability of research related to PVCO based transmitters in open literature, the proposed circuits are compared with previous works at different frequencies and in different technologies.

## 2. Design of PVCO Transmitters

Two circuits were designed, one operating at 433 MHz and the other at 2.4 GHz. Both circuits consist of a differential cross-coupled negative-*g_m_* VCO.

### 2.1. PVCO Design (433 MHz)

**Figure 2.**
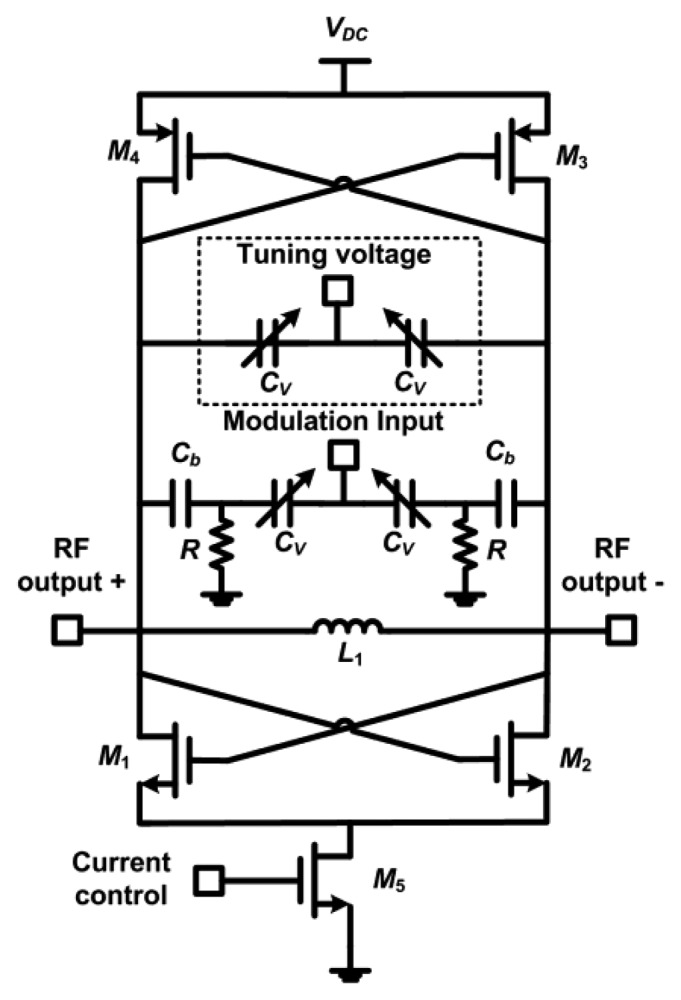
Basic schematic of the direct-modulation transmitter. The set of varactors shown within the dashed lines were only implemented in the 433 MHz design. Inductor *L_1_* is 18 nH in the 433 MHz design and 2.3 nH in the 2.4 GHz design.

Figure 2 shows the basic schematic of the proposed single stage direct-modulation transmitter. The tuning network consists of an LC-tank using inductor *L_1_* and a number of NMOS accumulation-mode varactors *C_V_* that have a variable capacitance of 1 to 3 pF. Inductor *L_1_* is 18 nH with a low quality-factor (*Q*) of 2, which has a major effect on the efficiency of the circuit and its phase noise performance. Since the operating frequency of this design is relatively low (433 MHz), it was possible to use 16 varactors in parallel to have a wider tuning range. The varactors were controlled through two different signals to have more flexibility in tuning the circuit and in applying the baseband modulation. Capacitors *C_b_* act as DC blocking capacitors with a value of 12 pF each and resistors *R*, each of 50 kΩ, provide a DC ground for biasing of the varactors. Each of the RF outputs goes to the 50 Ω load of the measurement equipment directly without a buffer. The loads will be replaced by the transmission antenna in the final system.

Transistors *M_1_*-*M_4_* form the cross coupled negative-*g_m_* differential pair. Each NMOS device has a total width of 200 μm using 80 fingers (finger width = 2.5 μm) and a length of 0.18 μm, while the PMOS devices have double the number of fingers. The sizes of these transistors are chosen based on a tradeoff between the required output power, operating frequency and achievable efficiency. The design equations can be found in [13]. Transistor *M_5_* acts as a current source that can be controlled in order to vary the transmitted power. This is a long channel device that uses 500 fingers with an aspect ratio of 1.25 mm/1 μm.

### 2.2. PVCO Design (2.4 GHz)

The 2.4 GHz PVCO design utilizes the same architecture of Figure 2; however, the set of varactors used for tuning (portion within the dashed lines) was removed and the total number of varactors used was 2. Inductor *L*_1_ in this design is 2.3 nH with a quality-factor (*Q*) of 8. In transistors *M*_1_-*M*_4_, the NMOS devices each have a total width of 175 μm using 70 fingers (finger width = 2.5 μm) and a length of 0.18 μm, while the PMOS devices have double the number of fingers. Transistor *M_5_* has the same aspect ratio as used in the 433 MHz design.

## 3. Design of PLL Transmitter

**Figure 3.**
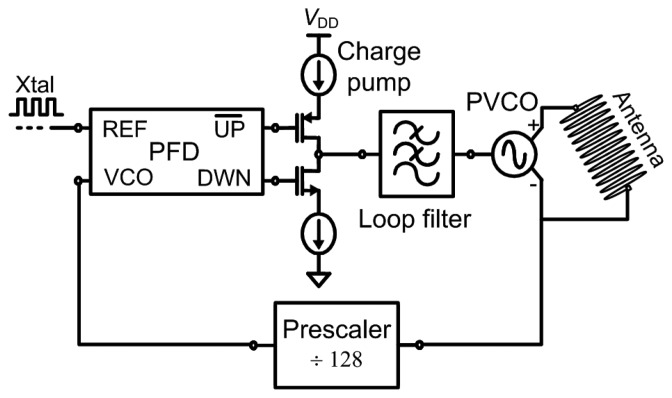
Block diagram of PLL transmitter. The antenna shown is not a part of the transmitter, it is an external component.

The basic block diagram of the PLL-based transmitter is shown in Figure 3. As the PVCO drives the antenna with the required output power at a frequency of 2.0–2.1 GHz, its frequency is divided by the prescaler, which is a divide-by-128 logic circuit and was implemented as a cascade of seven toggle flip-flops (TFFs). The divided frequency is then compared by the phase-frequency detector (PFD) to the external reference frequency coming from the crystal (Xtal), which should be in the range of 16 MHz. The output of the PFD will control the charge pump together with the loop filter in order to speedup or slowdown the PVCO until the output frequency and phase of the PVCO are locked to the Xtal reference. Both FSK and FM modulation can be applied by pulling the resonant frequency of the crystal, which would be considered indirect modulation of the VCO [14]. The important blocks of PLL transmitter are described in the following subsections.

### 3.1. PVCO Design (2.0 GHz)

**Figure 4.**
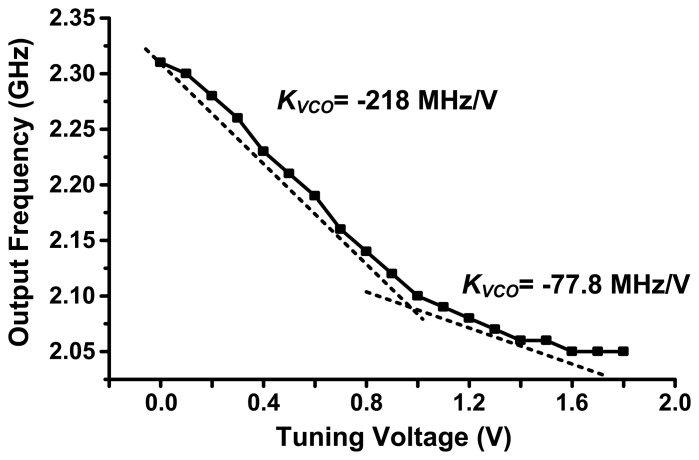
The simulated output frequency of oscillation as a function of the tuning voltage.

The 2.0 GHz PVCO design utilizes the same architecture of Figure 2; however, the portion within the dashed lines was removed. The NMOS and PMOS devices use the same dimensions as used in 2.4 GHz PVCO design. The simulated gain of the PVCO (*K_VCO_*) is −218 MHz/V in the tuning range from 0 to 1 V and −77.8 MHz/V in the tuning range from 1.0 to 1.8 V as shown in Figure 4.

### 3.2. Phase-Frequency Detector and Charge-Pump

The PFD is used to detect phase/frequency differences between the input signal coming from the PVCO and the reference signal coming from the crystal. The difference is translated into a proportional control signal that tunes the PVCO accordingly. There are many techniques used to provide such a function and the most commonly used is the PFD architecture since it increases the acquisition range and the lock speed of the PLL. Due to the finite speed of the PFD components, as the phase and frequency difference of the reference and PVCO signal approach zero, the output pulses do not approach zero linearly. Therefore, when the PLL is in the locked state, the control voltage can wander around the reference value resulting in a dead-zone, which can produce undesired spurs in the output spectrum. A charge pump is used after the PFD to charge or discharge the loop filter resulting in an increase or decrease in the output control voltage. The actual current-to-voltage conversion is done by the loop filter followed by the charge pump.

### 3.3. Loop Filter

**Figure 5.**
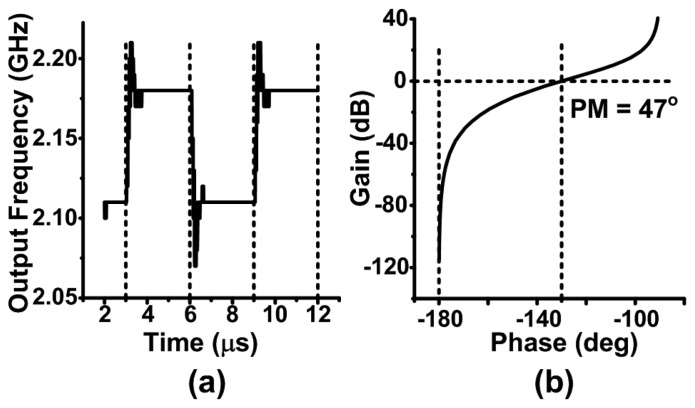
(**a**) Simulated response to step changes in the reference frequency showing a settling time less than 1.5 μs. (**b**) Nichols chart showing the stability of the system.

A third-order loop filter was designed in order to provide good filtering of the ripples in the VCO tuning voltage, while achieving a wide bandwidth for the PLL. It is very important to select the loop filter values properly. The design equations for a third-order loop filter can be found in [15]. Figure 5a shows the simulated PLL response to a number of steps in the input frequency, which shows a settling time less than 1.5 μs. The Nichols chart of the loop is shown in Figure 5b. A phase margin of 47 degrees was designed for, which is a good compromise between stability and settling time.

## 4. Measurement Results of PVCO Transmitters

**Figure 6.**
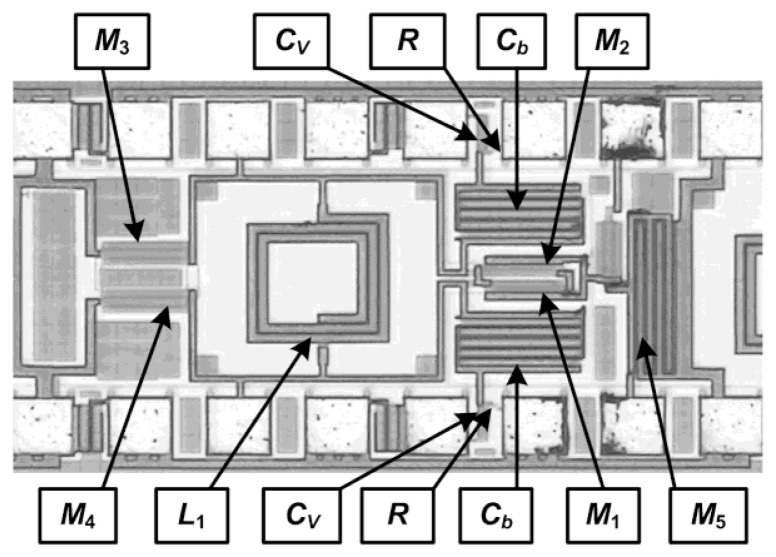
Photomicrograph of the fabricated 2.4 GHz direct-modulation transmitter.

All the circuits were fabricated in a 6-metal layer, 0.18 μm CMOS technology, with a 2 μm thick top-metal layer. Figure 6 shows a photomicrograph of the fabricated 2.4 GHz transmitter that occupies an area of only 0.6 mm^2^ including the pads. The inductors, all major interconnections and the RF pads were laid out using the top metal layer to minimize parasitic effects following the approach in [16]. The fabricated chips were tested on-wafer using RF probes of a ground-signal-ground (GSG) configuration for the RF signals and a single pad for DC connections. Measurements were performed by connecting one side of the differential output to the instrument and dummy-loading the other side with a 50 Ω load.

**Figure 7.**
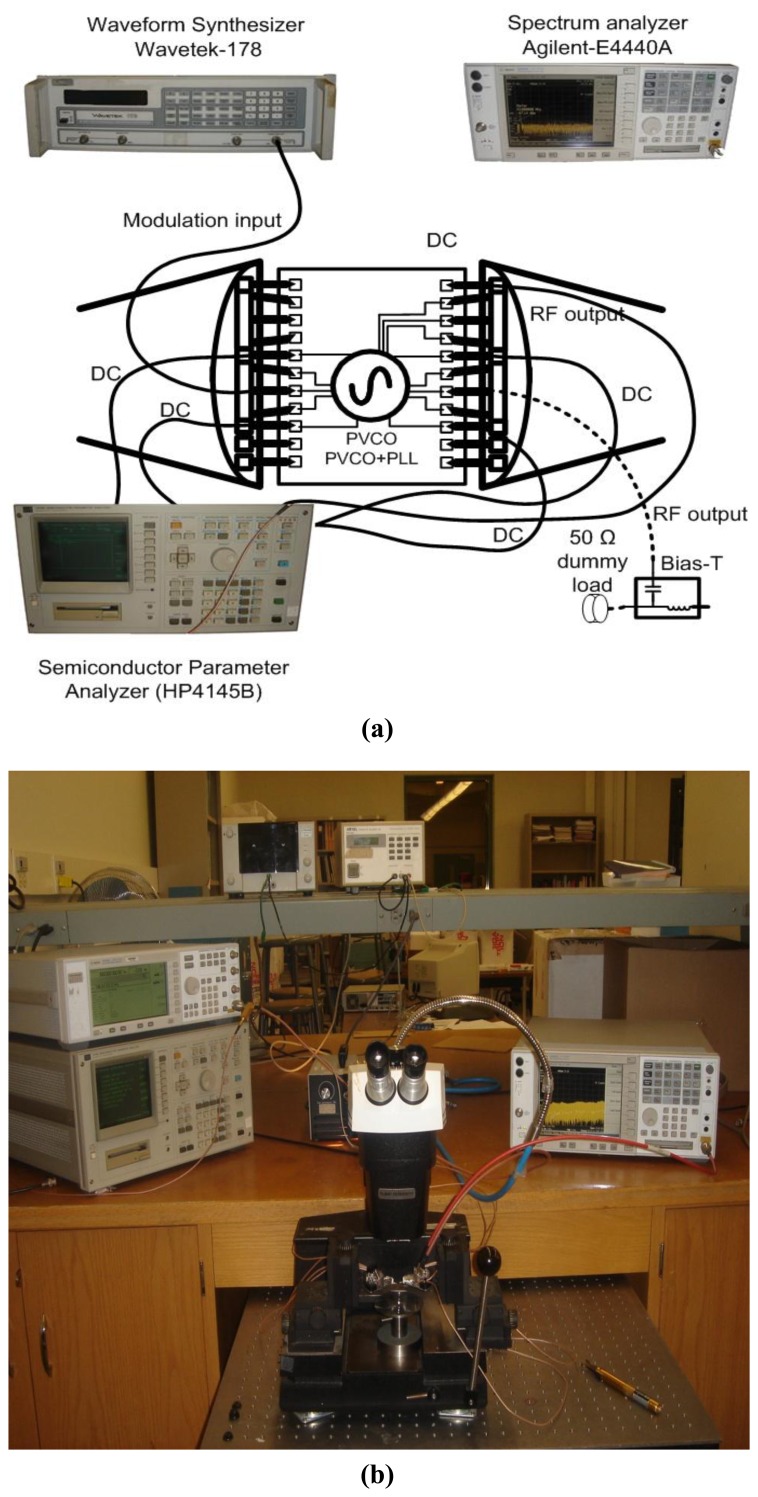
Experimental Setup used for the measurements of the transmitters showing the setup schematic in (**a**) and the photograph of the setup in (**b**).

The output signal was measured using an Agilent-E4440A spectrum analyzer and the baseband modulation was provided using a Wavetek-178 waveform synthesizer. An HP4145B semiconductor parameter analyzer was used to provide the biasing and to measure the DC power; however a DC battery box was used to bias the circuit for carrying out the phase noise measurements. This was done to avoid the increase in phase noise introduced by the output of the semiconductor parameter analyzer. The schematic and photograph of the actual experimental setup used for the measurement of direct-modulation transmitters are shown in Figure 7a and b, respectively.

### 4.1. Performance of 2.4 GHz PVCO Transmitter

**Figure 8.**
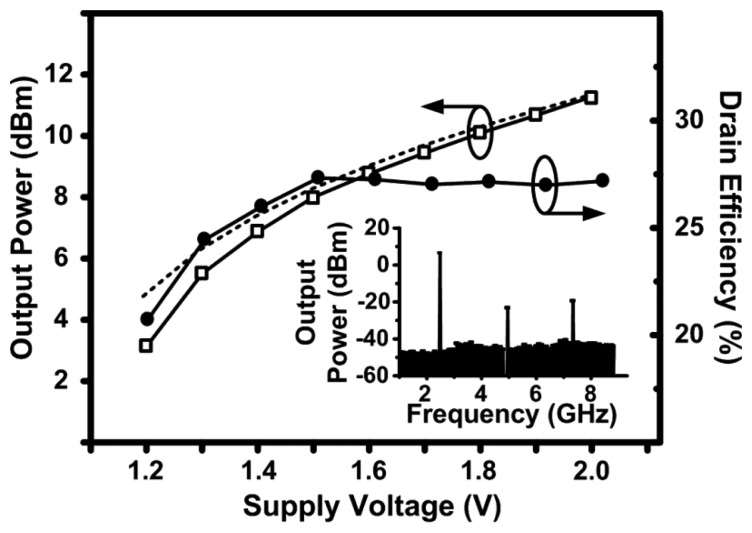
Measured output power and drain efficiency of the 2.4 GHz design as a function of the supply voltage. The dashed line shows the simulated output power and the inset figure shows the measured spectrum of the output signal at a supply voltage of 1.5 V.

Figure 8 shows the measured and simulated output power and the measured drain efficiency of the 2.4 GHz design as a function of the supply voltage. The drain efficiency is defined as the ratio of the output power delivered to the load to the DC power consumed from the supply. During this sweep, the tail current source (transistor *M*_5_ in Figure 2) was biased in the linear region to test the maximum effect of the supply voltage variation. The output power follows an expected square-law relationship with the supply voltage curve, however the efficiency appears to increase to a peak value of 27 % and then slightly decrease. The increase is due to varying the biasing of the MOSFET devices as the supply voltage varies, resulting in a variation in the transconductance of the device (*g_m_*). The MOSFET devices used in the cross-coupled VCO topology have equal drain and gate biasing and *g_m_* tends to increase rapidly with biasing to a peak maximum value, after which, it begins to decrease. As the supply voltage increases further, the gate biasing of the active devices increases resulting in a higher conduction period, which drops the efficiency such as in class-A amplifiers [17].

The power-added efficiency (PAE) in such a design is equal to the drain efficiency since the power gain can be considered infinite. This is due to the fact that the input power, which is required to drive the varactors by the digital baseband signal, is negligible. Figure 8 also shows the spectrum of the output signal. The second and third order harmonics are approximately 30 dB below the fundamental and no on-chip filter was used to suppress the higher order harmonics. An external narrow-band antenna can be used to suppress the higher-order harmonics [18–20], thus avoiding the need for an on-chip filter to maintain the achieved efficiency.

**Figure 9.**
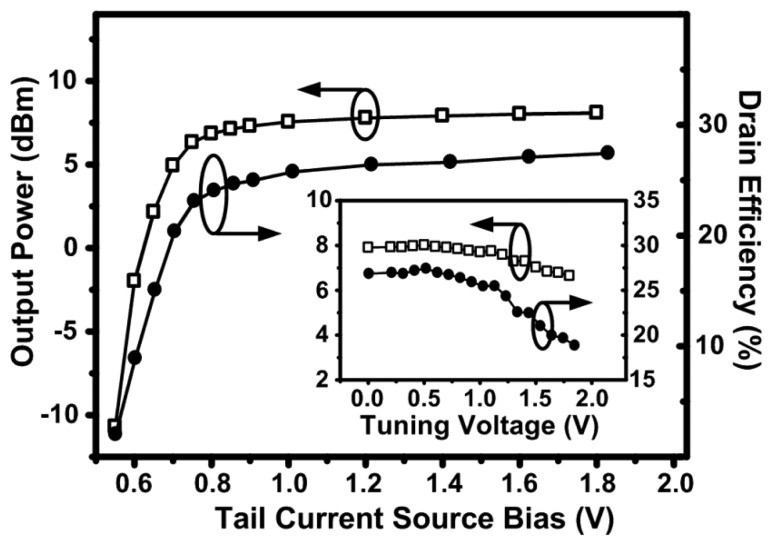
Measured output power and drain efficiency of the 2.4 GHz design as a function of the tail current source bias (transistor *M_5_* in Figure 2) and the tuning voltage (inset figure) applied to the varactors, at a supply voltage of 1.5 V.

Figure 9 shows the measured output power and drain efficiency of the 2.4 GHz design as a function of the tail current source biasing and the tuning voltage applied to the varactors at a supply voltage of 1.5 V. The gate biasing of the tail current source can be used to control the output power. As the tail current bias increases, the oscillator moves from the current limited regime to the voltage limited regime at a transition gate bias of 0.7 V. Increasing the gate bias of the tail current source no longer has an effect on the output power in the voltage limited regime; since the tail current source moves out of saturation and enters the linear region. The efficiency however, slightly increases since the resistance of the tail current source is reducing as *V_gs_* increases, causing less power loss in the active device. The output power slightly drops as the varactor tuning voltage increases since the quality-factor of the varactors goes down, which results in a drop in efficiency.

To improve the drain efficiency, several variations were investigated, which included changing the size of the filtering capacitors applied to the DC supply, using higher *Q*-factor varactors, using smaller DC-blocking capacitors, using a smaller bias resistor *R*, and finally using an inductor that is laid out over a deep *n*-well isolated substrate [10]. However, by increasing the DC supply filtering capacitors and reducing the size of the blocking capacitor, the drain efficiency was improved from 27% to 36% for the 2.4 GHz design.

**Figure 10.**
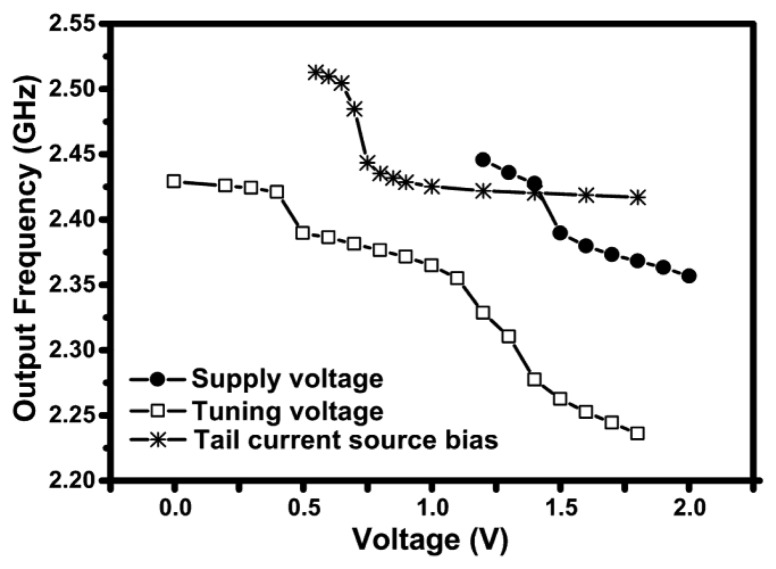
Measured frequency of oscillation of the 2.4 GHz design as a function the tail current source bias, supply voltage and tuning voltage. During the sweep of one bias voltage, the other two were kept constant at 1.5 V, except for the tuning voltage, which was kept constant at 0.5 V.

The output frequency of oscillation of the 2.4 GHz PVCO as a function of the tail current source bias, the supply voltage and the tuning voltage, is shown in Figure 10. The voltages were swept over the ranges for which the circuit oscillates. The circuit does not oscillate for supply voltages below 1.2 V due to the low gain and the stacked transistors in this topology. Low gate biases applied to the tail current source also cause the oscillation to stop, since the current flowing into the circuit will be too low, resulting again in a very low gain in the transistors. The output frequency is a strong function of the tail current source bias in the current limited regime; however, it is hardly affected by the tail current bias in the voltage limited regime since the DC current in the circuit no longer depends on the tail current source, as previously shown in Figure 9. The supply voltage was kept constant at 1.5 V as the tail current bias was varied. In case of the supply voltage sweep, the tail current bias was kept constant at a value of 1.5 V to keep the circuit operating in the voltage limited regime to enable us to see the effect of varying supply voltage, since its effect on the oscillation frequency is negligible in the current limited regime. In order to minimize the effects of voltage supply ripples, the circuit can be operated in the current limited mode.

Figure 10 shows that the output frequency is mostly affected by the tuning voltage, which gives a tuning range of 9% at about 0.2 GHz/V. A high tuning range is not desired in this circuit since the tuning voltage will only be used to modulate the output signal. The tuning curve exhibits a poor linearity over the whole range of the sweep since the device used is a MOS varactor, which is known to be very non-linear [21,22], especially in the depletion to accumulation transition region. However, since the modulation input will only vary the varactor voltage in the millivolt range, the modulation can be considered reliable within small ranges (0 V to 0.4 V or 0.5 V to 1 V) where the tuning curve is almost linear.

**Figure 11.**
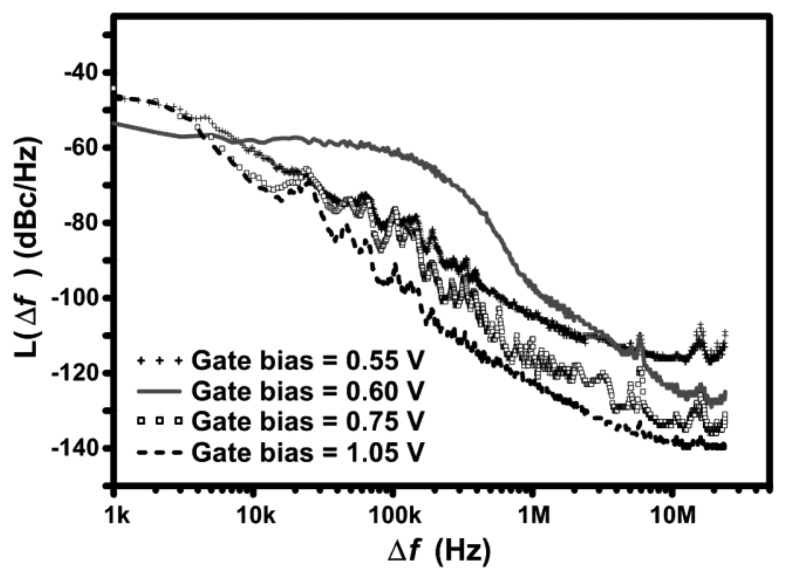
Measured phase noise of the 2.4 GHz design for various tail current source control voltages applied to transistor *M_5_*, at a supply voltage of 1.5 V.

The phase noise measurements of the 2.4 GHz design are shown in Figure 11 for various control voltages applied to the gate of the tail current source (transistor *M*_5_). The phase noise generally decreases as the circuit goes more into the voltage limited regime. Since oscillation frequency of the circuit in the current limited mode is very sensitive to the value of the biasing current, as previously shown in Figure 10, the slow random fluctuations in the tail current cause the frequency to jitter, resulting in phase noise.

Low-frequency flicker noise [23,24] generated by the tail current source upconverts into 1/*f*^3^ phase noise causing modulation of the biasing current and the amplitude of the output signal. The 1/*f*^2^ phase noise also reduces in the voltage limited mode since the current source is the main contributor of 1/*f*^2^ due to down-conversion of white channel noise at the current source where the frequency is double the output frequency [25]. The phase noise is not shown in Figure 11 for higher voltage levels since they have the same trend.

In Figure 11, the curve of 0.6 V control voltage stands out with an increase in 1/*f*^3^ phase noise. When looking carefully at the curves, the phase noise is low for both biasing points before and after the 0.6 V curve. Based on the results previously shown in Figure 9 and Figure 10, at low biases such as 0.55 V, the circuit is operating in the current limited mode. At higher gate biases above 0.7 V, the tail current source enters the linear region and loses its effect on the circuit, which makes the circuit operate in the voltage limited regime. At some point in between, the circuit is going through a transition from one mode to the other at which both modes can be competing, causing an increase in phase noise. An important point to note from this behavior is that such bias points should be avoided to improve the phase noise performance of the circuit and the tail current source should either be biased in weak inversion or biased with a high gate voltage, such that the device enters the triode mode.

**Figure 12.**
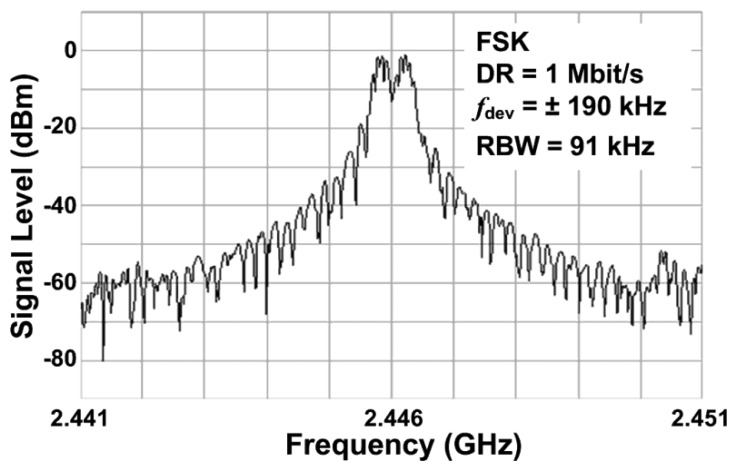
Measured output spectrum of the 2.4 GHz design, FSK modulated at 1 Mbit/s and ± 190 kHz frequency deviation, at a supply voltage of 1.5 V.

The 2.4 GHz design has a phase noise of -90 dBc/Hz at a 50 kHz offset and a phase noise of −122 dBc/Hz at a 1 MHz offset with a bias of 1.5 V applied to transistor *M*_5_, hence, operating in the voltage limited regime. Even though the proposed PVCO is fabricated in CMOS, it achieves phase noise comparable to low power CMOS designs and high power designs in other technologies [7,8,10,11]. Figure 12 shows the measured output power spectrum of the 2.4 GHz design in FSK mode taken from a single ended output at a supply voltage of 1.5 V. The two peaks are 380 kHz apart, modulated by a 1 MHz, 5 mV square wave applied to the varactors.

### 4.2. Performance of 433 MHz PVCO Transmitter

**Figure 13.**
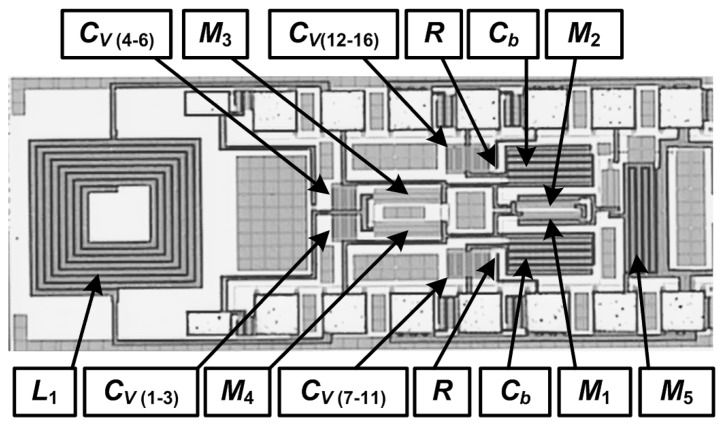
Photomicrograph of the fabricated 433 MHz direct-modulation transmitter.

**Figure 14.**
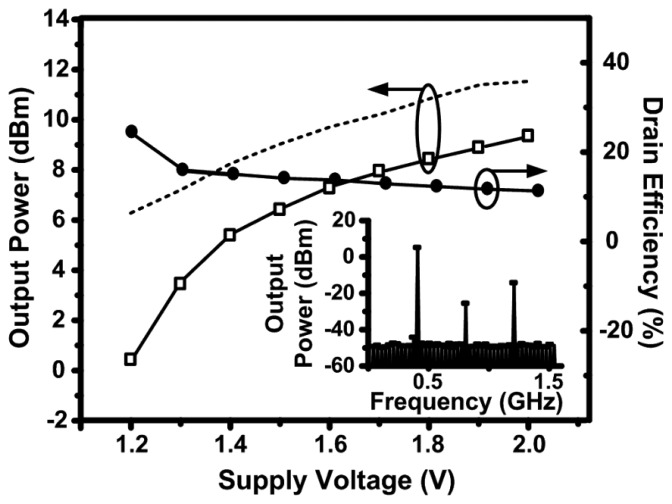
Measured output power and drain efficiency of the 433 MHz design as a function of the supply voltage. The dashed line shows the simulated output power and the inset figure shows the measured spectrum of the output signal at a supply voltage of 1.5 V.

Figure 13 shows a photomicrograph of the fabricated 433 MHz transmitter that occupies an area of only 0.9 mm^2^ including the pads with all components fully integrated. The increase in area compared to the 2.4 GHz design is due to the need for a larger inductor (18 nH), which was a 7.5 turn in this case. Figure 14 shows the measured and simulated output power and the measured drain efficiency of the 433 MHz design as a function of the supply voltage. The sweep was done over the voltage range for which the circuit oscillates. Since the output voltage swing increases with a higher supply voltage, the output power also increases. The discrepancy between the measured and simulated output power in this design is higher than the 2.4 GHz design, since the quality factor of the inductor estimated by the equivalent circuit model was not accurate at this frequency. At a supply voltage of 1.2 V, the drain efficiency had already reached its peak value and started to slightly degrade since larger transistors, which have a higher *g_m_*, were used in this design than the 2.4 GHz design.

**Figure 15.**
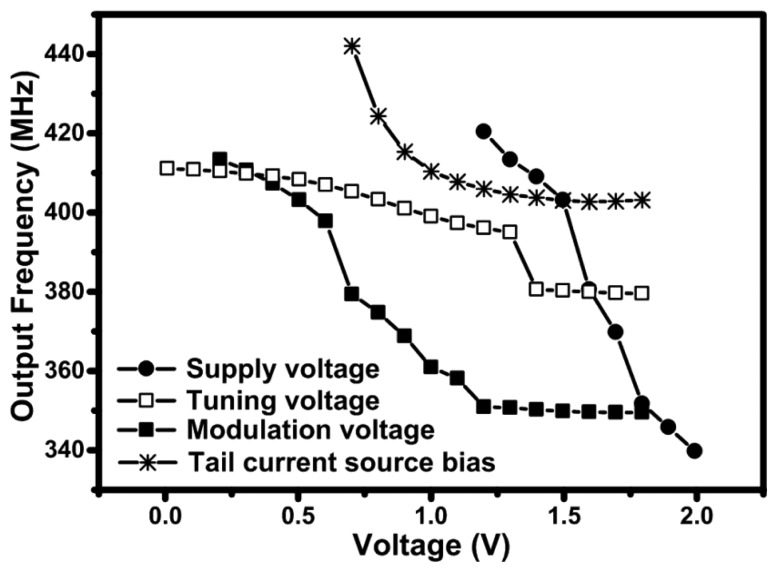
Measured output frequency of oscillation of the 433 MHz design as a function the supply voltage, tuning voltage, modulation voltage and tail current source bias. During the sweep of one bias voltage, the other three were kept constant at 1.5 V, except for the tuning voltage and modulation voltage, which were kept constant at 0.5 V.

The output frequency of oscillation of the 433 MHz design is shown in Figure 15 as a function of the supply voltage, the tuning voltage, the modulation voltage and the tail current source bias. Based on the modulation voltage signal only, the circuit has a tuning range of 16 %, which is larger than the 2.4 GHz design due to the large number of varactors used. The circuit can be tuned to operate in both the 433 MHz ISM band and the European 405 MHz medical implantable communication systems (MICS) band.

The phase noise performance of the 433 MHz design has a similar behavior to that of the 2.4 GHz design, as shown in Figure 11 previously. The circuit has a phase noise of −84 dBc/Hz at a 50 kHz offset and a phase noise of −120 dBc/Hz at a 1 MHz offset with a bias of 1.5 V applied to transistor *M*_5_. The phase noise is slightly degraded when compared to the 2.4 GHz design, mainly due to the lower quality factor of the large 18 nH on-chip inductor used in the 433 MHz design.

### 4.3. Performance Comparison of PVCO Transmitters

The well-established figure-of-merit (FoM) used in oscillator designs [3] is not suitable for comparing PVCO since the FoM does not include a term for the transmitted output power. The following equation shows a modified FoM that was used to compare the performance of PVCOs:

(1) $$\text{FoM}={P_{\text{out}}|}_{\text{dBm}}-10\times \log\left(\overset{P_{dc}}{\;}/\underset{1mW}{\;}\right)+20\times \log\left(\frac{\omega_{0}}{\Delta \omega }\right)-L(\Delta \omega )-20\times \log Q$$

where, *P_out_* is the RF output power and *P_dc_* is the DC power consumption of the oscillator normalized to 1 mW. The ratio of *P_out_* to *P_dc_* is a measure of the drain efficiency of the PVCO. Parameter *ω*_0_ is the angular frequency of oscillation of the oscillator, Δ*ω* is the offset frequency at which the phase noise *L*(Δ*ω*) is measured and *Q* is the quality-factor of the inductor used in the tank circuit.

Table 1 summarizes the performance comparison of designed PVCO direct-modulation transmitters with previously published VCO implementations using the proposed FoM [7,8,11,26–28]. The work presented in this paper shows that the CMOS PVCO can achieve acceptable performance level comparable to other technologies, while at the same time providing high level of integration and low-cost.

## 5. Measurement Results of PLL Transmitter

**Figure 16.**
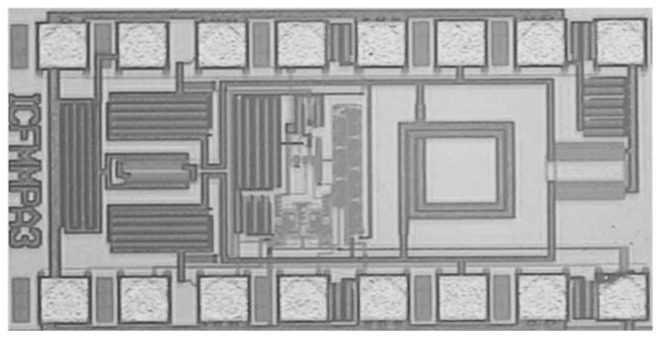
Photomicrograph of the fabricated PLL transmitter.

Figure 16 shows a photomicrograph of the fabricated transmitter that occupies an area of only 0.77 mm^2^, including the pads [17]. The inductor and all major interconnections were laid out using the top metal layer to minimize parasitic effects and only the top metal layer was used for RF pads to minimize parasitic capacitances. Separate biasing pads and lines were used for digital and analog circuits and the logic circuits were laid out in a deep-n-well to isolate their substrate from the substrate of the high power VCO signals following the layout considerations from [13,16]. The same experimental set-up was used for the measurement of PLL transmitter as shown in Figure 7.

The circuit was tested with a supply voltage of 1.8 V for both the digital and analog parts. When applying a 0.55 V rms, 15.8 MHz reference signal, the measured output power is 9 mW with an output frequency of 2.0224 GHz. The DC current consumed by the PLL is 2 mA, while the current consumed by the PVCO is 24 mA. This results in a total drain efficiency of 19%. The PLL has a lock range of 128 MHz and a capture range of 104 MHz.

**Figure 17.**
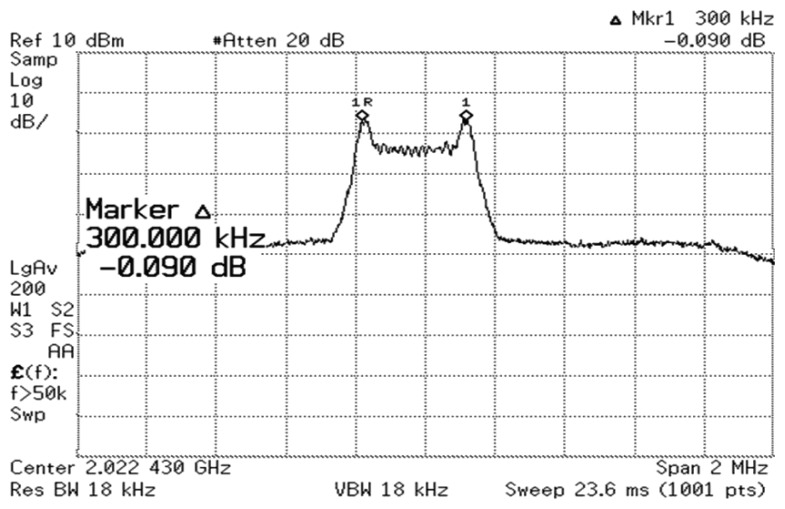
The measured output spectrum with a 10 kbps FSK modulated input and ±1.172 kHz frequency deviation, at a supply voltage of 1.8 V.

**Figure 18.**
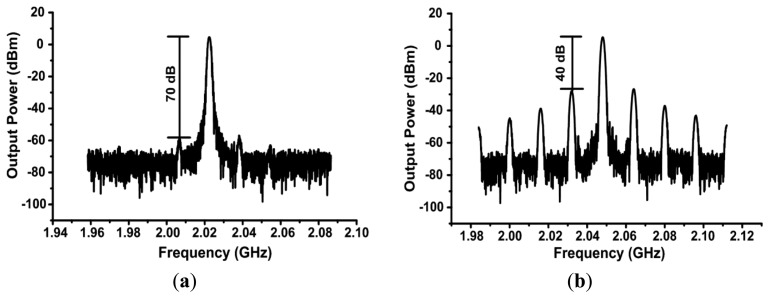
The measured output spectrum of the PLL transmitter showing the (**a**) best case spurs. (**b**) worst case spurs.

An FSK modulated input was applied at the reference point with a modulation rate of 10 kbps and a deviation frequency of 1.172 kHz. The measured output spectrum, averaged over 200 samples, is shown in Figure 17. The figure shows a frequency deviation of 150 kHz from the center, which is equivalent to 128 times the deviation applied to the input at the reference point. Figure 18a shows the measured output spectrum with the best case largest spurs at 70 dBc, whereas Figure 18b shows the worst case spurs at 40 dBc.

The change from Figure 18a to Figure 18b was done by increasing the reference frequency from 15.8 MHz to 16 MHz, which corresponds to a change in the output frequency from 2.0224 GHz to 2.048 GHz respectively. These spurs can be attributed to the mismatch between the NMOS and the PMOS devices in the charge pump current or even substrate coupling due to the high power signals of the VCO. However, the values of the spurs are more than 40 dB below the carrier, so they will not have a significant effect on the system's performance for such applications. Table 2 summarizes the performance evaluation of 2.4 GHz and 433 MHz PVCO transmitters with a 2.0 GHz PLL transmitter.

## 6. Conclusions

In this paper, design and implementation of 2.4 GHz, 433 MHz PVCO and 2.0 GHz PLL based direct-modulation transmitters are presented for short-range wireless applications. All three RF transmitters were fabricated in a standard CMOS technology. Performance evaluation of PVCO transmitters with the previously reported implementations is done using the proposed figure-of-merit. Measurement results of the 2.4 GHz transmitter demonstrate an improved drain efficiency of 36%. 2.4 GHz and 433 MHz PVCO transmitters deliver an output power of 8 dBm with a phase noise of −122 dBc/Hz at a 1 MHz offset and 6.5 dBm with a phase noise of −120 dBc/Hz at a 1 MHz offset respectively. The PLL transmitter measurement results demonstrate an output power of 9 mW with a total DC current of 26 mA from a 1.8 V supply. The transmitter die areas are 0.6 mm^2^, 0.9 mm^2^ and 0.77 mm^2^ for 2.4 GHz, 433 MHz and 2.0 GHz designs respectively. The results demonstrate that the proposed circuits can achieve acceptable performance level with considerable reduction of transmitter die area and power consumption, leading to simpler and more efficient designs that are suitable for emerging low-power applications such as WSNs and WBANs.

## Figures and Tables

**Table 1.** Performance comparison with previously published VCO transmitters.

**Ref.**	[7]	[8]	[11]	[26]	[27]	[28]	**This work**		
Tech.	0.35 μm CMOS	GaN FET	0.6 μm GaAs MESFET	0.18 μm CMOS	0.18 μm CMOS	0.18 μm CMOS	**0.18μm CMOS**		
*f* (GHz)	2.4	3.0	3.8	2.4	2.4	2.4	**0.43**	**2.4**	**2.4 (Improved)**
*P*_out_ (dBm)	6	34	3	7	10.2	16.1	**6.5**	**8**	**7.6**
*Eff*.(%)	-	23	37	-	30	-	**14**	**27**	**36**
*Quality factor (Q)*	6.5	5 (assumed)	20	10 (assumed)	10	14.1	**2**	**8**	**8**
*L*(Δ*Ω*) (dBc/Hz)	−103 "@100kHz	−80 "@100kHz	−132 "@5MHz	−108 "@5MHz	−118 "@1MHz	−108 "@1MHz	−**120** "@**1MHz**	−**122** "@**1MHz**	−**122** "@**1MHz**
*FoM* (dB)	167	150	159	137	160	152	**158**	**166**	**167**

**Table 2.** Performance evaluation of direct-modulation RF transmitters

	**433 MHz (PVCO Design)**	**2.4 GHz (PVCO Design)**	**2.4 GHz (Improved PVCO Design)**	**2.0 GHz (Integrated PVCO-PLL Design)**
Output Power	6.5 dBm	8 dBm	7.6 dBm	9.5 dBm
Current Dissipation	20.8 mA	15.4 mA	10.7 mA	26 mA
Power Dissipation	31.2 mW	23.1 mW	16.1 mW	46.8 mW
Supply Voltage	1.5 V	1.5 V	1.5 V	1.8 V
FSK Fdev	±15 kHz	±190 kHz	±190 kHz	1.172 kHz
Data Rate	500 kbps	1 Mbps	1 Mbps	10 kbps
Phase Noise "@ 1 MHz	−120 dBc/Hz	−122 dBc/Hz	−122 dBc/Hz	-
Sidebands	−48 dBc	−34 dBc	−34 dBc	−40 dBc
†Die Area	0.9 mm^2^	0.6 mm^2^	0.6 mm^2^	0.77 mm^2^

† All designs were fabricated and fully integrated in a standard 0.18 μm CMOS 1-poly 6-metal technology provided by TSMC through the Canadian Microelectronics Corporation (CMC).
